# Identification of stable housekeeping genes for induced pluripotent stem cells and -derived endothelial cells for drug testing

**DOI:** 10.1038/s41598-022-20435-w

**Published:** 2022-09-28

**Authors:** Sheena L. M. Ong, Hans J. Baelde, David G. P. van IJzendoorn, Judith V. M. G. Bovée, Karoly Szuhai

**Affiliations:** 1 Department of Pathology, Leiden University Medical Center, Leiden, The Netherlands; 2 Department of Pathology, Stanford University School of Medicine, Stanford, CA USA; 3 Department of Cell and Chemical Biology, Leiden University Medical Center, Einthovenweg 20, 2333 ZC Leiden, The Netherlands

**Keywords:** Biological techniques, Stem cells

## Abstract

There are no validated housekeeping genes in induced pluripotent stem cells (iPSC) and derived endothelial iPSC (iPSC-EC). Thus a comparison of gene expression levels is less reliable, especially during drug treatments. Here, we utilized transcriptome sequencing data of iPSC and iPSC-EC with or without CRISPR-Cas9 induced translocation to identify a panel of 15 candidate housekeeping genes. For comparison, five commonly used housekeeping genes (*B2M*, *GAPDH*, *GUSB*, *HMBS*, and *HPRT1*) were included in the study. The panel of 20 candidate genes were investigated for their stability as reference genes. This panel was analyzed and ranked based on stability using five algorithms, *delta-Ct*, *bestkeeper*, *geNorm*, *Normfinder*, and *Reffinder*. Based on the comprehensive ranking of *Reffinder*, the stability of the top two genes—*RPL36AL* and *TMBIM6*, and the bottom two genes—*UBA1* and *B2M*, were further studied in iPSC-EC with and without genetic manipulation, and after treatment with telatinib. Using quantitative reverse-transcriptase polymerase chain reaction (qRT-PCR), it was shown that gene expression of the top two housekeeping genes, *RPL36AL* and *TMBIM6,* remained stable during drug treatment. We identified a panel of housekeeping genes that could be utilized in various conditions using iPSC and iPSC-derived endothelial cells as well as genetically modified iPSC for drug treatment.

## Introduction

Induced pluripotent stem cells (iPSCs) are broadly used in scientific research to generate different cell types in the context of basic or applied research to develop potential therapies^1–3^, for example, the generation of isogenic iPSC models, either patient-derived or healthy donor-derived, to model pathogenic conditions and drug screening^4^. The advancement in genetic manipulation and iPSC differentiation methods allows investigators to gain new insight into the functional consequences of specific molecular alterations. We previously generated in vitro model using iPSCs to study vascular tumor pseudomyogenic hemangioendothelioma (PHE). Rearrangement involving *FOSB* is a characteristic of *PHE*, the most common binding partners are *SERPINE1* and *ACTB*^5,6^. To mimic PHE, we introduced chromosomal translocation t(7;19)(q22;q13) leading to the *SERPINE1-FOSB* fusion using iPSC model^7^.

PHE is a rare, locally aggressive, rarely metastasizing endothelial neoplasm that occurs in bone and soft tissue in young adults with a strong male predominance^8^. The tumour is often multifocal and locally aggressive. It forms rarely metastasis and is classified clinically as an intermediate category^9^. After surgical resection, approximately 60% of patients experience local recurrences or develop additional tumors in the same anatomical location^9^. Chemotherapy or radiotherapy is generally given to treat patients with multifocal disease. Recently, the use of systemic targeted therapies using mTOR inhibition (sirolimus, everolimus, and rapamycin) or telatinib, a VEGFR1–4/PDGFRA multi-tyrosine kinase inhibitor has shown clinical benefit in reported cases^10–13^. Histologically, these tumors consist of loose fascicles of plump spindle cells with abundant and brightly eosinophilic cytoplasm^8^. Immunohistochemically, there is a characteristic expression of ERG, CD31, and keratin AE1/AE3, while CD34 and desmin are negative and INI1 retained^8^, highlighting its vascular differentiation despite the lack of vasoformation. The *SERPINE1-FOSB* leads to overexpression of FOSB at RNA and protein as the fusion transcript is driven by the *SERPINE1* promoter region^14,15^.

Given that the presumed cell of origin of vascular tumors is endothelial cells, the iPSC^SERPINE1-FOSB^ cells towards the endothelial lineage (iPSC-EC^SERPINE1-FOSB^) to facilitate the functional evaluation of specific genetic alterations^7^. This model may shed light on the involved pathways and facilitate the identification of targeted drugs for treatment. Previously, a PHE patient was shown to have undergone complete remission during treatment with telatinib, possibly through the inhibition of FLT1, FLT4, and PDGFRA signaling^13^. However, the effect of telatinib in treating PHE has yet to be validated. QRT-PCR expression analysis could be used to monitor cellular reaction to drug treatments, such as telatinib, in iPSC-EC^SERPINE1-FOSB^ and detect the expression of *FOSB*, *SERPINE1*, and the *SERPINE1-FOSB* fusion transcript.

QRT-PCR is a broadly used technique that allows the relative quantification of gene expression. For reliable measurement and comparison, the identification and inclusion of stably-expressed housekeeping genes are needed. Examples of frequently used housekeeping genes are *GAPDH*, *B2M*, and *HPRT*^16^. However, these genes are differentially expressed in mammalian tissue types^17^. During reprogramming of human iPSC, expression of *GAPDH* was relatively stable compared to other common housekeeping genes such as *ACTB*, *B2M*, and *HPRT*^18^. Likewise in iPSC and neurons derived from iPSC, *GAPDH* is one of the most stably expressed genes^19^. However, an extensive study that compared *GAPDH* expression in 72 different human tissues revealed that the expression of *GAPDH* varies across tissue types^20^. Housekeeping genes are extensively used across various cell lines, though these housekeeping genes may not necessarily be stable in all cell lines^19,20^. Definitive identification of a stable housekeeping gene panel is laborious work. There is a need for validated reference genes in iPSC and iPSC-EC.

This study aimed to identify a set of housekeeping genes that remains stable between iPSC and iPSC-EC with and without gene manipulation and upon drug treatment. We used the transcriptome data from our previously iPSC and iPSC-EC samples with and without a gene fusion^7^. Five widely used algorithms, *delta-Ct*, *geNorm*, *NormFinder*, *BestKeeper,* and *RefFinder*^21–25^ were utilized to identify and verify the best reference housekeeping gene set. Here, we identified a panel of 15 candidate genes and compared their stability to the five most commonly used housekeeping genes, *B2M, GAPDH, GUSB*, *HMBS*, and *HPRT1,* in this field. From a total of 20 genes, *RPL36AL*, *TMBIM6*, *MORF4L2*, *HPRT1*, and *SLC25A3* were superior to *B2M* and *GAPDH* based on a comprehensive housekeeping gene ranking. The top two ranked housekeeping genes, *RPL36AL* and *TMBIM6* remain stable in genetically manipulated and endothelial differentiated cells during drug treatment.

## Results

### Housekeeping genes and their primer specificity

Using our transcriptome sequencing data from iPSC and iPSC-EC with and without *SERPINE1-FOSB* fusion^7^, we ranked gene expressions according to the lowest standard deviation (SD) and coefficient of variation (CV) of their fragments per kilobase of exon per million mapped fragments (FPKM) value. FPKM is a unit of expression which estimate gene expression based on transcriptome sequencing data^26^. We subsequently listed 15 candidate genes with an FPKM value of more than 100 and determined their suitability as housekeeping genes (Table 1). Of these 15 candidate genes, the *YWHAZ* gene is frequently used as a housekeeping gene and ranked first based on our ranking metrics^27^. We added five common housekeeping genes, *B2M, GAPDH, GUSB*, *HMBS*, and *HPRT1,* to compare their stability with the 15 candidate genes. The specificity and the amplification range of the primers that were designed for these 20 genes were analysed using a dilution of the template cDNA and using melt runs to identify by-products. The designed primers were specific and efficient when tested over a dilution range, as represented by their respective melting curve shown in Supp Fig. 1 and coefficient of R^2^ value between 0.98 to 1.00 (Supp Table 2).

**Table 1:** Transcriptome values of housekeeping genes.

Rank (lowest SD and CV)	Symbol	iPSC-EC^SERPINE1-FOSB^			iPSC-EC^WT^			iPSC^SERPINE1-FOSB^			iPSC^WT^			Average	SD	CV
		Sample #1 FPKM	Sample #2 FPKM	Sample #3 FPKM	Sample #1 FPKM	Sample #2 FPKM	Sample #3 FPKM	Sample #1 FPKM	Sample #2 FPKM	Sample #3 FPKM	Sample #1 FPKM	Sample #2 FPKM	Sample #3 FPKM			
1	YWHAZ	302	308	294	315	315	313	311	326	324	295	344	296	312	15	0
**2**	**GUSB**	**29**	**30**	**28**	**32**	**29**	**31**	**25**	**21**	**20**	**22**	**18**	**24**	**26**	**11**	**0**
3	ATP5F1C	152	160	145	156	149	150	145	142	153	145	151	129	148	8	0
4	MORF4L2	117	115	117	122	123	119	132	127	138	129	127	115	123	7	0
5	NDUFB10	117	131	110	114	115	108	113	112	99	107	122	111	113	8	0
6	RPN2	144	120	123	130	124	127	138	146	144	139	145	132	134	10	0
7	UBA52	576	661	535	605	541	600	618	678	640	659	635	638	616	46	0
8	PRELID1	191	203	171	169	176	176	205	204	181	181	182	166	184	14	0
9	UBA1	146	131	133	124	129	132	141	133	138	159	134	158	138	11	0
10	TMBIM6	201	216	203	232	229	215	188	199	205	182	204	183	205	16	0
11	PFDN5	140	159	140	153	149	148	124	133	123	145	130	142	141	11	0
12	PRKCSH	150	144	137	128	131	141	119	126	113	134	126	139	132	11	0
13	RPL36AL	232	279	232	259	248	252	271	271	267	230	264	209	251	21	0
14	SLC25A3	229	251	201	203	212	222	220	244	254	214	232	199	223	19	0
15	GANAB	182	168	178	178	173	177	154	144	143	159	154	153	164	14	0
**16**	**GAPDH**	**2276**	**2411**	**2015**	**1895**	**2075**	**2117**	**3741**	**3676**	**3535**	**4426**	**3404**	**4225**	**1829**	**817**	**0**
17	COX7A2	114	148	110	144	132	146	124	131	130	131	124	128	130	12	0
**18**	**B2M**	**412**	**504**	**410**	**510**	**431**	**392**	**52**	**55**	**61**	**48**	**46**	**50**	**382**	**167**	**0**
**19**	**HMBS**	**5**	**6**	**6**	**5**	**4**	**5**	**25**	**24**	**26**	**22**	**20**	**19**	**7**	**5**	**1**
**20**	**HPRT1**	**10**	**12**	**10**	**10**	**11**	**9**	**25**	**25**	**24**	**19**	**19**	**13**	**12**	**4**	**3**

Common housekeeping genes that were added into our study are highlighted in bold.

### Stability of reference genes using various algorithms

**Figure 1:**
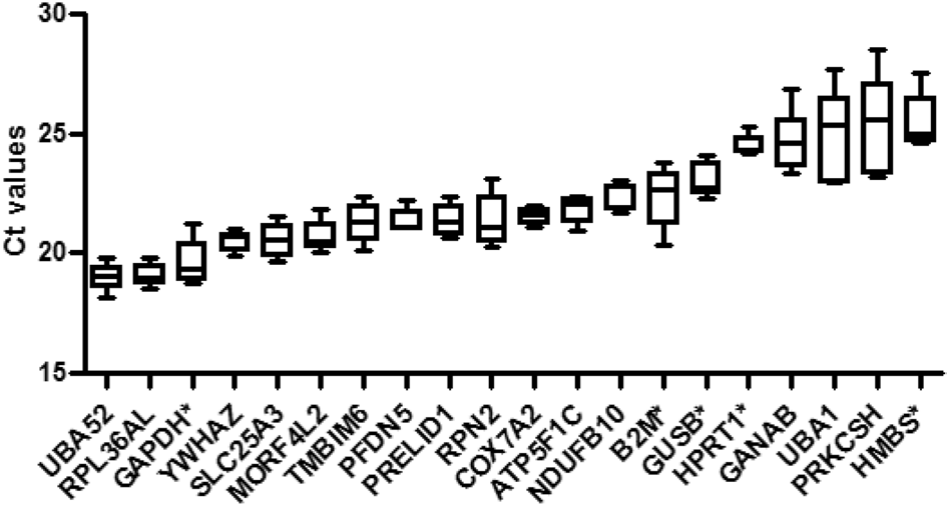
Mean Ct values. Mean Ct values of iPSC^WT^, iPSCS^SERPINE1-FOSB^, iPSC-EC^WT^ of each housekeeping gene were shown in a box-and-whisker plot and sorted from the lowest (left) to the highest (right). The five added common housekeeping genes are denoted with an asterisk. The whiskers represent SD of nine samples, three samples per cell line (iPSC^WT^, iPSCS^SERPINE1-FOSB^, and iPSC-EC^WT^).

Five different, commonly used algorithms (*delta-Ct*, *Bestkeeper*, *geNorm*, *Normfinder*, and *Reffinder*) were tested on iPSC^WT^, iPSC-EC^WT^, and iPSC-EC^SERPINE1-FOSB^ to identify the best matching housekeeping gene set using qRT-PCR. The data generated in the five algorithms were from three biological runs in technical triplicates. The expression of the 20 housekeeping genes is represented in Fig. 1. The mean Ct values range from 18 to 25.5 cycles. *PRKCSH* and *HMBS* showed the lowest expression levels, both with a mean Ct value of 25.5, while *UBA52* and *RPL36AL* displayed the highest expression levels with mean Ct values of 19.0 and 19.1, respectively (Supp Table 1).

#### Delta-Ct

The *delta-Ct* method determines the stability of candidate genes by comparing the relative expression of candidate genes among the samples^21^. The two most stable genes based on *delta-Ct* method are *YWHAZ* and *COX7A2,* with corresponding mean SD values of 0.15 and 0.16, while the least stable genes were *PRKCSH* and *UBA1* with mean SD values of 0.39 and 0.41, respectively. Of the five common housekeeping genes included in the study, only *HPRT1* falls in the top 10 stable mean SD values based on *delta-Ct*.

#### Bestkeeper

*Bestkeeper* evaluates candidate genes and ranks based on the lowest SD and CV. *COX7A2* and *UBA52* have the lowest variations in gene expression with corresponding CV values of 1.3% and 1.5% (Table 2). We observed that the lower expressed genes, such as *UBA1* and *PRKCSH*, have higher variation with a CV value of CP 3.6% and 4.5%, respectively. Only one commonly used housekeeping gene, *HPRT1*, was ranked third in this algorithm. *GUSB*, *GAPDH*, *HMBS*, and *B2M* remained low in the ranking, with a CV value of CP between 2.4% to 3.9%.

**Table 2:** *Bestkeeper* housekeeping gene ranking sorted according to their SD and CV% values and their crossing point (CP) .

HKG	COX7A2	UBA52	HPRT1	SLC25A3	TMBIM6	PRELID1	RPL36AL	ATP5F1C	PFDN5	YWHAZ	RPN2	NDUFB10	GUSB	MORF4L2	GAPDH	GANAB	HMBS	B2M	UBA1	PRKCSH
n	9	9	9	9	9	9	9	9	9	9	9	9	9	9	9	9	9	9	9	9
geo Mean [CP]	21.5	19.7	24.5	21.0	21.6	21.8	19.5	21.8	21.7	20.3	21.8	22.6	23.0	21.2	19.6	24.7	25.5	22.2	25.5	25.8
ar Mean [CP]	21.5	19.7	25.5	21.0	21.6	21.8	19.5	21.8	21.7	20.3	21.8	22.6	23.1	21.2	19.6	24.8	25.6	22.2	25.6	25.8
Min [CP]	21.1	18.9	24.2	20.4	21.1	21.2	18.9	21.0	21.0	19.4	21.1	21.7	22.3	20.3	18.8	23.3	24.7	20.4	24.2	24.0
Max [CP]	22.0	20.2	25.3	21.6	22.4	22.4	20.0	22.3	22.2	21.0	23.1	23.2	24.1	21.9	21.2	26.9	27.5	23.8	27.7	28.5
Std dev [± CP]	0.3	0.3	0.3	0.3	0.3	0.3	0.4	0.4	0.4	0.5	0.5	0.6	0,6	0.6	0.7	0.9	0.9	0.9	0.9	1.2
CV [%CP]	1.3	1.5	1.3	1.5	1.6	1.6	2.0	1.9	1.9	2.5	2.5	2.5	2.4	2.7	3.6	3.4	3.4	3.9	3.6	4.5
Rank	1	2	3	4	5	6	7	8	9	10	11	12	13	14	15	16	17	18	19	20

#### geNorm

**Figure 2:**
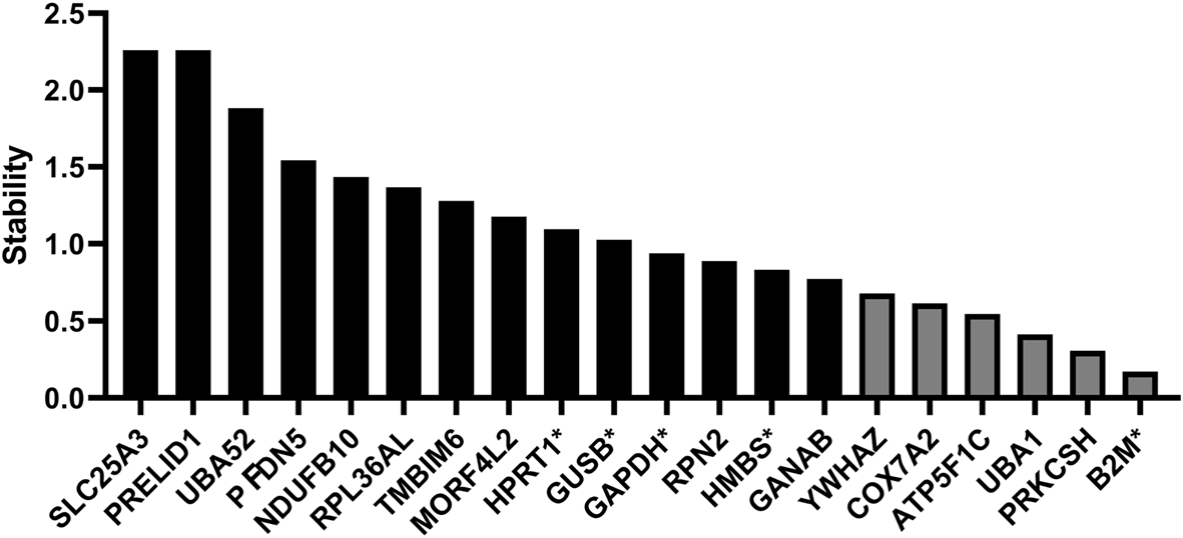
*geNorm* ranking of stability M value. Housekeeping genes are plotted based on their stability M value from the highest (left) to the lowest (right). Black bars are ideal housekeeping genes and grey bars are acceptable housekeeping genes. The five added common housekeeping genes are denoted with an asterisk. Note: this tool is not calculating SD.

*geNorm* measures the stability value (M) by calculating the pairwise expression ratio for each candidate gene against all the other genes^24,28^. Based on the *geNorm* ranking of M value, 14 genes were evaluated to be ideal housekeeping genes and six as acceptable. The genes with the highest stability M value (of 2.26) are *SLC25A3* and *PRELID1* (Fig. 2). The six genes that were designated as “acceptable”: *YWHAZ*, *COX7A2*, *ATP5F1C*, *UBA1*, *PRKCSH*, and *B2M*. The other four commonly used housekeeping genes, *HPRT1*, *GUSB*, *GAPDH*, and *HMBS* were classified as ideal.

#### NormFinder

**Figure 3:**
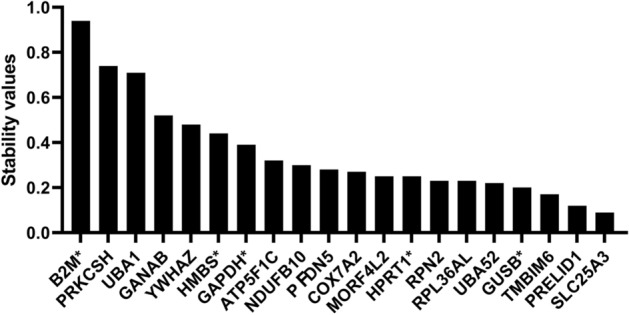
*NormFinder* ranking based on stability value. Housekeeping genes are plotted based on their stability value from the least (left) to the most stable (right). The five added common housekeeping genes are denoted with an asterisk.

*NormFinder* measures the intra- and intergroup variation to calculate the stability of candidate genes^23^. Similar to *geNorm* analysis, the two most stable genes identified by *Normfinder* are *SLC25A3* and *PRELID1*, and the two least stable genes are *PRKSCH* and *B2M* (Fig. 3). The most stable genes, *SLC25A3* and *PRELID1* have the lowest stability value of 0.09 and 0.12. The least stable genes, *PRKSCH* and *B2M* have high stability values of 0.74 and 0.94. Among the common housekeeping genes, *GUSB* ranked fairly high in *NormFinder* according to its stability value of 0.20. *HPRT1* was also ranked fairly, in the 8th position, with a stability value of 0.25. The other common housekeeping genes, *GAPDH*, *HMBS*, and *B2M*, were ranked 14th, 15th, and 20th, respectively. *NormFinder* algorithms calculated that the two genes that gave the best stability in our panel were *HPRT1* and *RPN2,* each with a stability value of 0.07.

#### RefFinder

*RefFinder* utilizes the four other algorithms to evaluate and rank candidate genes by assigning appropriate weights to candidate genes and calculating the geometric mean of their weights for the final ranking^25^. The comprehensive ranking generated by *RefFinder* ranked *RPL36AL* as the most stable gene with the lowest geometric mean of 2.91 (Table 3). *TMBIM6* was ranked second with the same geometric mean as *RPL36AL*. The top five genes were *RPL36AL*, *TMBIM6*, *MORF4L2*, *HPRT1*, and *SLC25A3,* respectively. In all five algorithms, *PRKCSH*, *UBA1*, and *B2M* were the least stable housekeeping genes.

**Table 3:** Housekeeping gene ranking of all five algorithms.

Gene	*Delta-Ct*		*Bestkeeper*		*geNorm*		*NormFinder*		*RefFinder*	
	Mean SD	Rank	SD	Rank	M value	Rank	Stability	Rank	Geomean	Comprehensive ranking
RPL36AL	0.19	4	0.39	7	1.37	6	0.23	6	2.91	1
TMBIM6	0.25	12	0.34	5	1.28	7	0.17	3	2.91	2
MORF4L2	0.21	6	0.57	14	1.18	8	0.25	8	4.21	3
HPRT1	0.22	8	0.31	3	1.10	9	0.25	9	4.76	4
SLC25A3	0.24	11	0.31	4	2.26	1	0.09	1	5.36	5
GANAB	0.34	17	0.85	16	0.77	14	0.52	17	5.69	6
RPN2	0.29	15	0.54	11	0.89	12	0.23	7	5.92	7
GUSB	0.28	14	0.56	13	1.03	10	0.20	4	6.96	8
PFDN5	0.20	5	0.42	9	1.54	4	0.28	11	6.98	9
COX7A2	0.16	2	0.27	1	0.62	16	0.27	10	7.36	10
UBA52	0.22	7	0.29	2	1.88	3	0.22	5	8.15	11
PRELID1	0.24	10	0.34	6	2.26	2	0.12	2	8.71	12
YWHAZ	0.15	1	0.50	10	0.68	15	0.48	16	10.53	13
NDUFB10	0.23	9	0.56	12	1.43	5	0.30	12	11.74	14
GAPDH	0.32	16	0.71	15	0.94	11	0.39	14	13.69	15
ATP5F1C	0.18	3	0.42	8	0.55	17	0.32	13	14.89	16
HMBS	0.35	18	0.86	17	0.83	13	0.44	15	15.21	17
PRKCSH	0.39	19	1.16	20	0.31	19	0.74	19	18.24	18
UBA1	0.41	20	0.92	19	0.41	18	0.71	18	19.25	19
B2M	0.27	13	0.87	18	0.17	20	0.94	20	19.48	20

### Stability of housekeeping genes during telatinib treatment

**Figure 4:**
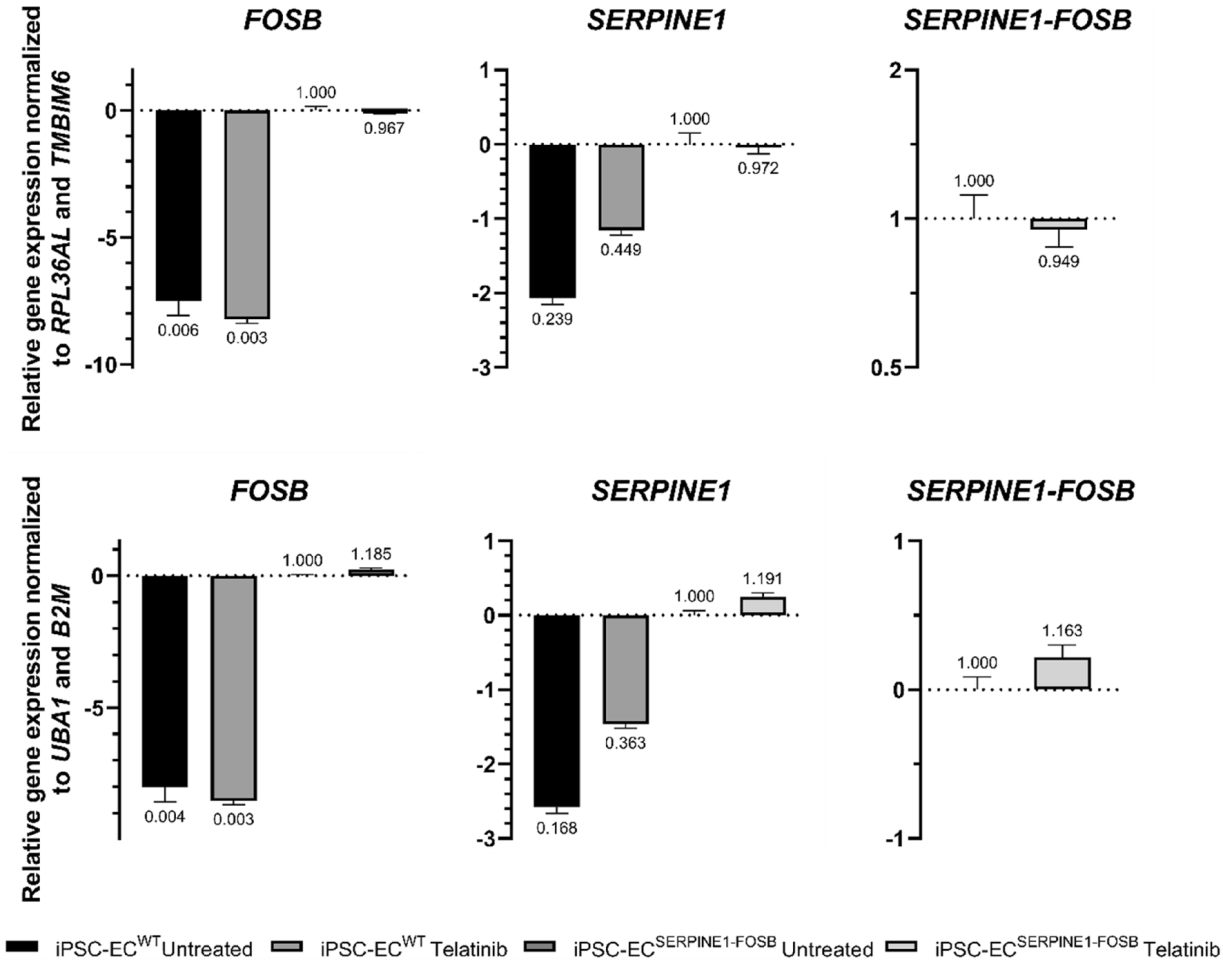
Gene expression of telatinib-treated cells. Relative to iPSC-EC^SERPINE1-FOSB^ untreated, the log scale expression of *FOSB*, *SERPINE1* or *SERPINE1-FOSB* were normalized to the top two, *RPL36AL* and *TMBIMB*, or bottom two, *UBA1* and *B2M*, housekeeping genes of telatinib treated or untreated iPSC-EC^WT^ and iPSC-EC^SERPINE1-FOSB^ samples. Three biological samples of each treatment were pooled together and run qRT-PCR in technical duplicates.

Using a single housekeeping gene for normalization could lead to relatively large errors, so we used a combination of two housekeeping genes for further studies^16,24^. Based on the comprehensive ranking of candidate housekeeping genes, we selected the top two genes, *RPL36AL* and *TMBIM6*, and the bottom two genes, *UBA1* and *B2M*, for further analysis of their gene expression stability during drug treatment with telatinib. QRT-PCR data generated are from biological duplicates and technical triplicates. The relative expression of *FOSB*, *SERPINE1*, *SERPINE1*-*FOSB* was normalized to the top or bottom two housekeeping genes (Fig. 4). When the expression of *FOSB* and *SERPINE1* was normalized to housekeeping genes *RPL36AL* and *TMBIM6*, telatinib treated iPSC-EC^SERPINE1-FOSB^ showed a slight reduction in expression as compared to iPSC-EC^WT^. However, when *FOSB* and *SERPINE1* expression were normalized to *UBA1* and *B2M*, telatinib treated iPSC-EC^SERPINE1-FOSB^ showed a slight increase in expression compared to iPSC-EC^WT^. In addition, we observed that *SERPINE1*-*FOSB* expression was reduced in telatinib treated iPSC-EC^SERPINE1-FOSB^ compared to untreated iPSC-EC^SERPINE1-FOSB^ when normalized to most ideal selected genes while the expression increased when normalized to least ideal selected genes. Of note, the significance level of the relative normalized expression of *SERPINE1*-*FOSB* between the top and bottom selected housekeeping gene is p < 0.4.

## Discussion

In this study, we aimed to identify a set of housekeeping genes that remain stable in iPSC and iPSC-EC with and without genetic manipulation or drug treatment for qRT-PCR. A previously established transcriptome sequencing data set of iPSC, iPSC-EC with and without CRISPR-Cas9 induced translocation was used to identify stable housekeeping genes. Next to the identified new housekeeping genes, we aimed to compare these with a commonly used housekeeping gene panel and showed that most of the new housekeeping genes were more stable than the commonly used housekeeping genes. Multiple studies have investigated candidate reference genes in iPSCs or ECs^18,29–33^, during reprogramming and differentiation^18,33^. In addition, various publications reported a selection of housekeeping genes in endothelial cells, such as microvascular EC^29^, human retinal EC^31^, and human blood–brain barrier EC^32^. The candidate reference genes examined in these studies were among the commonly used housekeeping genes in our study, including *B2M*, *GAPDH*, *GUSB*, *HPRT1*, *HMBS*, and *YWHAZ*. A potential drawback of the use of common housekeeping genes is their variable expression across different tissue types^16,17,20,34,35^.

From our transcriptome sequencing analysis, 14 of 15 genes were not validated previously as reference genes in the context of iPSC and derived EC. Intriguingly, these 14 genes were also identified in two independent studies through gene expression data from EST (expressed sequence tag), SAGE (series analysis of gene expression), and/or microarray that suggested their potential as candidate reference genes^36,37^. Some of these novel candidate reference genes showed better stability in various human frozen tissues and cell lines as compared to common housekeeping genes such as *B2M*, *HMBS*, and *GAPDH*^36^. In our study of 20 housekeeping genes, *PRKCSH*, *UBA1*, and *B2M* were ranked the three lowest of all housekeeping genes validated. *B2M* was previously investigated as a candidate reference gene when examined in reprogrammed iPSC or EC and was ranked between four to eight of a panel of 10 housekeeping genes^18,29,38^. Likewise in the iPSC and derived neurons study, *GAPDH* and *HMBS* were ranked among the top five of 16 reference genes^19^. In our study using all five algorithms, *GAPDH* was ranked between 11 to 15 of 20, while *HMBS* was ranked between 13 to 17 of 20. This suggests that using other reference genes, such as *RPL36AL* or *TMBIM6*, could yield better results than the common housekeeping genes. However, the stability of these reference genes in other types of differentiated iPSC should be validated.

Next to tissue type differences, variation in the expression of common housekeeping genes was observed within the same cell line when exposed to different conditions^30,31,39^. For example, statin-treated HUVEC cells showed that *HPRT1* and *YWHAZ* were considered the most suitable reference genes^39^. However, in homocysteine-treated HUVEC cells, *YWHAZ* was not stably expressed and ranked seven of eight^31^. In addition, hydrogen peroxide-treated HUVEC showed *HPRT1* was a less reliable reference gene with a ranking of 11 of 15^30^. These studies stressed the importance of validating reliable reference genes in different physiological conditions. In telatinib-treated cells, the *SERPINE1*-*FOSB* expression showed a reduction in expression, when normalized to the top two housekeeping genes—*RPL36AL* and *TMBIM6*. However, when normalized to the bottom two housekeeping genes—*UBA1* and *B2M*, an increase in expression was observed. The discrepancy in the expression of *SERPINE1*-*FOSB* between the top and bottom selected housekeeping genes was concerning and further supports the importance of verifying the stability of housekeeping genes during drug treatment.

In this study, the validation of housekeeping genes should fit into three criteria, 1. Stable expression in both iPSC and iPSC-EC, 2. Stable expression in wild-type and mutant lines, and 3. Stable expression during treatment with telatinib. As such, in our study, we found that *RPL36AL*, *TMBIM6*, *MORF4L2*, *HPRT1*, and *SLC25A3* were ranked the top five genes. Common housekeeping genes such as *GAPDH*, *HMBS*, and *B2M* are less stably expressed in our panel of cell lines. The top two ranked genes, *RPL36AL* and *TMBIM6*, remained stable in both iPSC and iPSC-EC with and without *SERPINE1*-*FOSB* translocation and during drug treatment with telatinib, making them ideal housekeeping genes.

We identified a panel of HKGs that could be utilized in various conditions using iPSC and iPSC-derived endothelial cells as well as genetically modified iPSC cells for drug treatment.

## Materials and methods

### Cell lines and cell culture and drug treatment

The human iPSC line LUMC0054iCTRL (http://hpscreg.eu/cell-line/LUMCi001-A)^40^ was cultured on Vitronectin XF™ (STEMCELL technologies, 07180) coated plates in TeSR™-E8™ Kit for ESC/iPSC Maintenance (STEMCELL technologies, 05990) according to manufacturers’ instructions. The *SERPINE1-FOSB* translocated iPSC (iPSC^SERPINE1-FOSB^) was generated in our previous study^7^. iPSCs were differentiated into endothelial cells in three independent biological replicates as previously described^41^. Endothelial cell-differentiated iPSC were treated with or without 5 µM of telatinib for 14 h in serum-starved condition followed by four hours of serum stimulation. Each treatment condition were carried out in biological triplicates but pooled together when harvested for further analysis.

### RNA isolation and quantitative real-time-polymearse chain reaction

Cells were homogenized and RNA was isolated using TRIzol (Ambion, 15596018) and DNase I treated and purified according to the RNeasy kit (Qiagen, 74104). First-strand cDNA was synthesized using the iScript™ cDNA Synthesis Kit (Bio-rad, 1708891). qRT-PCR reactions were carried out with iQ™ SYBR^®^ Green Supermix (Bio-rad, 1708886). The samples that were used to determine the stability of reference genes are iPSC^WT^, iPSC^SERPINE1-FOSB^, and iPSC-EC^WT^. For iPSC^WT^ and iPSC^SERPINE1-FOSB^, RNAs at three different passages were harvested. While for iPSC-EC^WT^, cells from three independent differentiation setups were harvested for analysis. These samples (biological replicates in triplicate) were run in triplicate in PCR for analysis (technical replicates). Untreated and drug-treated samples of iPSC-EC^WT^ and iPSC-EC^SERPINE1-FOSB^ of a single passage number were run in technical duplicate and used to analyze the stability of selected reference genes. All of the samples were run in a two-step PCR setting with an annealing temperature of 60 °C for 45 cycles. An overview of the 20 housekeeping genes and their primer sequences is shown in Supp Table 2. The primers used to amplify *FOSB*, *SERPINE1*, and *SERPINE1*-*FOSB* are listed in Supp Table 3. The amplification and melt curve of the designed primers was examined over a dilution range (1, 1/4, 1/16, 1/64, and 1/256) for their specificity.

### Transcriptome data identification of housekeeping gene candidates

Previously generated and published transcriptome data (accession PRJNA448372) was used to select stable genes across various conditions^7^. Normalized FPKM gene expressions of induced pluripotent stem cells and derived endothelial cells of WT and *SERPINE1-FOSB* translocation were obtained and sorted according to the lowest SD and CV. The top 15 expressed genes with an FPKM value of > 100 were selected. In addition, independent of the ranking results, five commonly used housekeeping genes such as *GUSB*, *GAPDH*, *B2M*, *HMBS*, and *HPRT1* were included for comparison.

### Statistical analysis

The same generated qRT-PCR data were utilized in five algorithms to assess the expression stability of 20 reference genes, *delta-Ct*^21^, *Bestkeeper*^22^, *geNorm*^24^, *NormFinder*^23^, and *RefFinder*^25^. Three of the algorithms, *delta-Ct*, *Bestkeeper*, and *RefFinder* use untransformed Ct values as input. The remaining two algorithms, *geNorm* and *NormFinder*, the average delta Ct values were used. The *delta-Ct* method compares the mean SD of reference genes and the calculated values were exported from CFX Maestro software. The excel based software, *Bestkeeper*, was manually extended to accommodate 20 reference genes and the Ct values were used for analysis^22^. *Bestkeeper* tabulates the geometric mean, arithmetic mean, the minimal and maximal Ct values and the percentage of CV based on the crossing point values of each reference gene as opposed to other excel based software, *NormFinder*, in which the delta Ct values were input and the ANOVA-based mathematical analysis was used to calculate expression stability values^23^. *geNorm* was tabulated through the reference gene selector tool in the CFX Maestro software that calculates expression stability M values per reference genes as detailed in Vandesompele 2002. In both *geNorm* and *NormFinder*, a low stability value indicates more stable expressed gene^23,24^. We obtained the comprehensive ranking using a web-based tool, *RefFinder*, that compares and ranks the reference genes based on four algorithms, *geNorm*, *NormFinder*, *Bestkeeper* and *delta-CT methods*^25^. Statistical analysis comparing the expression of *FOSB*, *SERPINE1*, and *SERPINE1-FOSB* between the top and bottom two housekeeping genes was carried out using a *t* test.

## Supplementary Information

Supplementary Information.

## Data Availability

The datasets generated and analysed during the current study are available from the corresponding author on request.
