# Author Correction: Identification of stable housekeeping genes for induced pluripotent stem cells and -derived endothelial cells for drug testing

**DOI:** 10.1038/s41598-025-04721-x

**Published:** 2025-06-16

**Authors:** Sheena L. M. Ong, Hans J. Baelde, David G. P. van IJzendoorn, Judith V. M. G. Bovée, Karoly Szuhai

**Affiliations:** 1 Department of Pathology, Leiden University Medical Center, Leiden, The Netherlands; 2 Department of Pathology, Stanford University School of Medicine, Stanford, CA USA; 3 Department of Cell and Chemical Biology, Leiden University Medical Center, Einthovenweg 20, 2333 ZC Leiden, The Netherlands

Correction to: *Scientific Reports* 10.1038/s41598-022-20435-w, published online 28 September 2022

The original version of this Article contained an error in the gene name PFDN5, which was incorrectly given as PRDN5 in six instances.

As a result, Table 2

**Table:** 

HKG	COX7A2	UBA52	HPRT1	SLC25A3	TMBIM6	PRELID1	RPL36AL	ATP5F1C	PRDN5

now reads,

**Table:** 

HKG	COX7A2	UBA52	HPRT1	SLC25A3	TMBIM6	PRELID1	RPL36AL	ATP5F1C	PFDN5

Table 3

**Table:** 

Gene	*Delta-Ct*		*Bestkeeper*		*geNorm*		*NormFinder*		*RefFinder*	
	Mean SD	Rank	SD	Rank	M value	Rank	Stability	Rank	Geomean	Comprehensive ranking
RPL36AL	0.19	4	0.39	7	1.37	6	0.23	6	2.91	1
TMBIM6	0.25	12	0.34	5	1.28	7	0.17	3	2.91	2
MORF4L2	0.21	6	0.57	14	1.18	8	0.25	8	4.21	3
HPRT1	0.22	8	0.31	3	1.10	9	0.25	9	4.76	4
SLC25A3	0.24	11	0.31	4	2.26	1	0.09	1	5.36	5
GANAB	0.34	17	0.85	16	0.77	14	0.52	17	5.69	6
RPN2	0.29	15	0.54	11	0.89	12	0.23	7	5.92	7
GUSB	0.28	14	0.56	13	1.03	10	0.20	4	6.96	8
PRDN5	0.20	5	0.42	9	1.54	4	0.28	11	6.98	9

now reads,

**Table:** 

Gene	*Delta-Ct*		*Bestkeeper*		*geNorm*		*NormFinder*		*RefFinder*	
	Mean SD	Rank	SD	Rank	M value	Rank	Stability	Rank	Geomean	Comprehensive ranking
RPL36AL	0.19	4	0.39	7	1.37	6	0.23	6	2.91	1
TMBIM6	0.25	12	0.34	5	1.28	7	0.17	3	2.91	2
MORF4L2	0.21	6	0.57	14	1.18	8	0.25	8	4.21	3
HPRT1	0.22	8	0.31	3	1.10	9	0.25	9	4.76	4
SLC25A3	0.24	11	0.31	4	2.26	1	0.09	1	5.36	5
GANAB	0.34	17	0.85	16	0.77	14	0.52	17	5.69	6
RPN2	0.29	15	0.54	11	0.89	12	0.23	7	5.92	7
GUSB	0.28	14	0.56	13	1.03	10	0.20	4	6.96	8
PFDN5	0.20	5	0.42	9	1.54	4	0.28	11	6.98	9

In addition, Figures 1, 2, and 3 contained the incorrect gene in their X-axes, and in the Supplementary Information in Table S1. The incorrect files appear below.

**Fig. 1:**
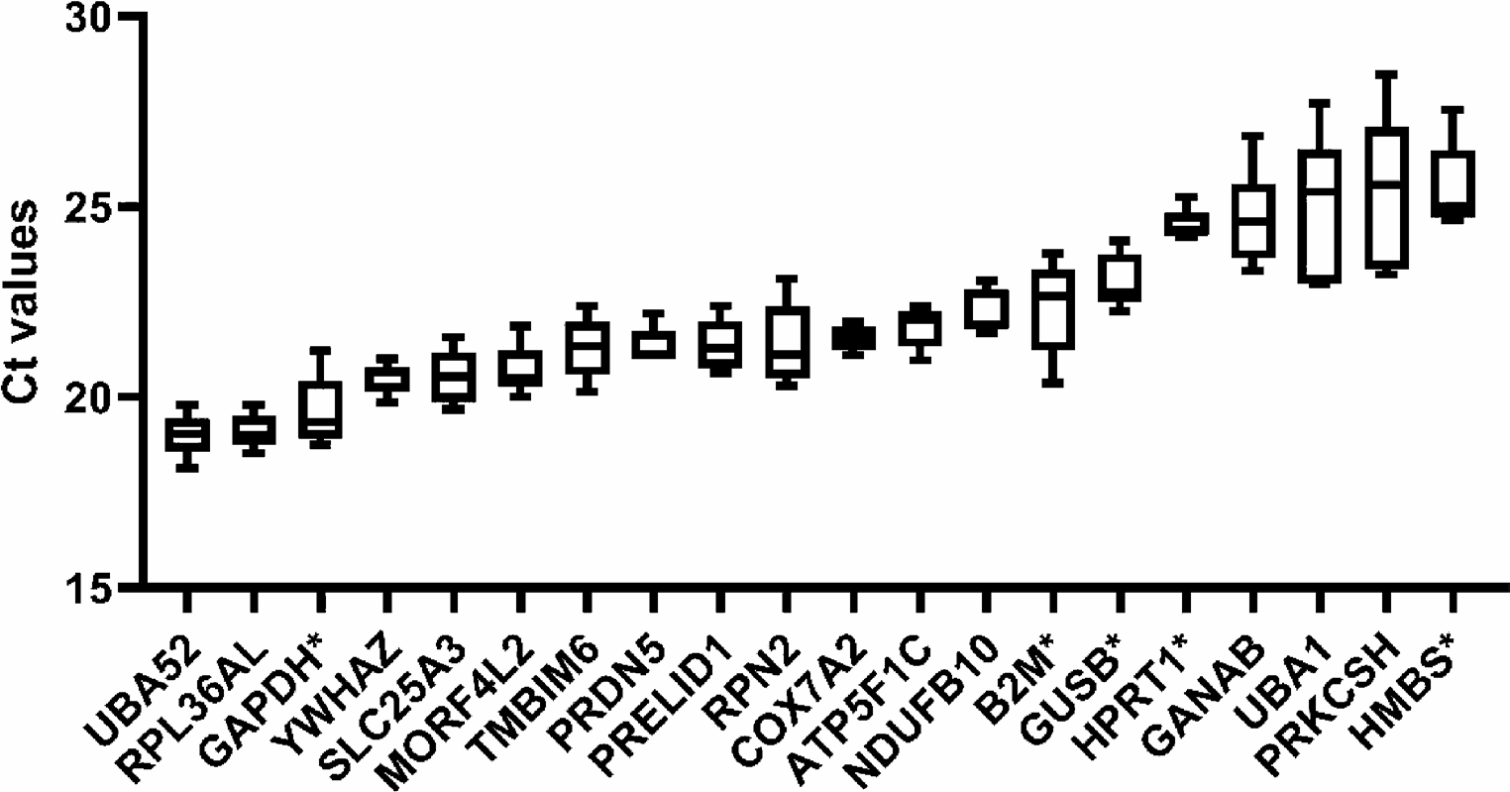
Mean Ct values. Mean Ct values of iPSC^WT^, iPSCS^SERPINE1-FOSB^, iPSC-EC^WT^ of each housekeeping gene were shown in a box-and-whisker plot and sorted from the lowest (left) to the highest (right). The five added common housekeeping genes are denoted with an asterisk. The whiskers represent SD of nine samples, three samples per cell line (iPSC^WT^, iPSCS^SERPINE1-FOSB^, and iPSC-EC^WT^).

**Fig. 2:**
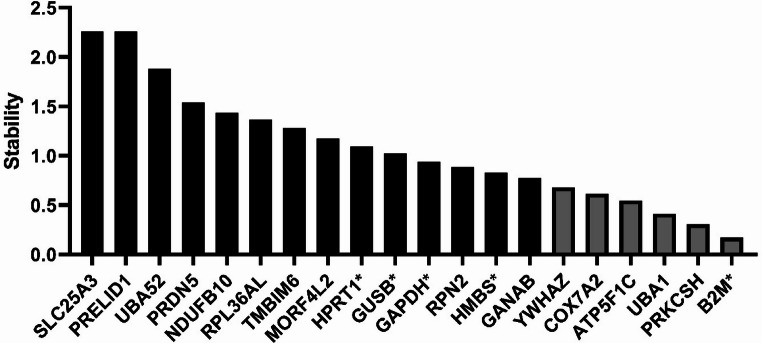
*geNorm* ranking of stability M value. Housekeeping genes are plotted based on their stability M value from the highest (left) to the lowest (right). Black bars are ideal housekeeping genes and grey bars are acceptable housekeeping genes. The five added common housekeeping genes are denoted with an asterisk. Note: this tool is not calculating SD.

**Fig. 3:**
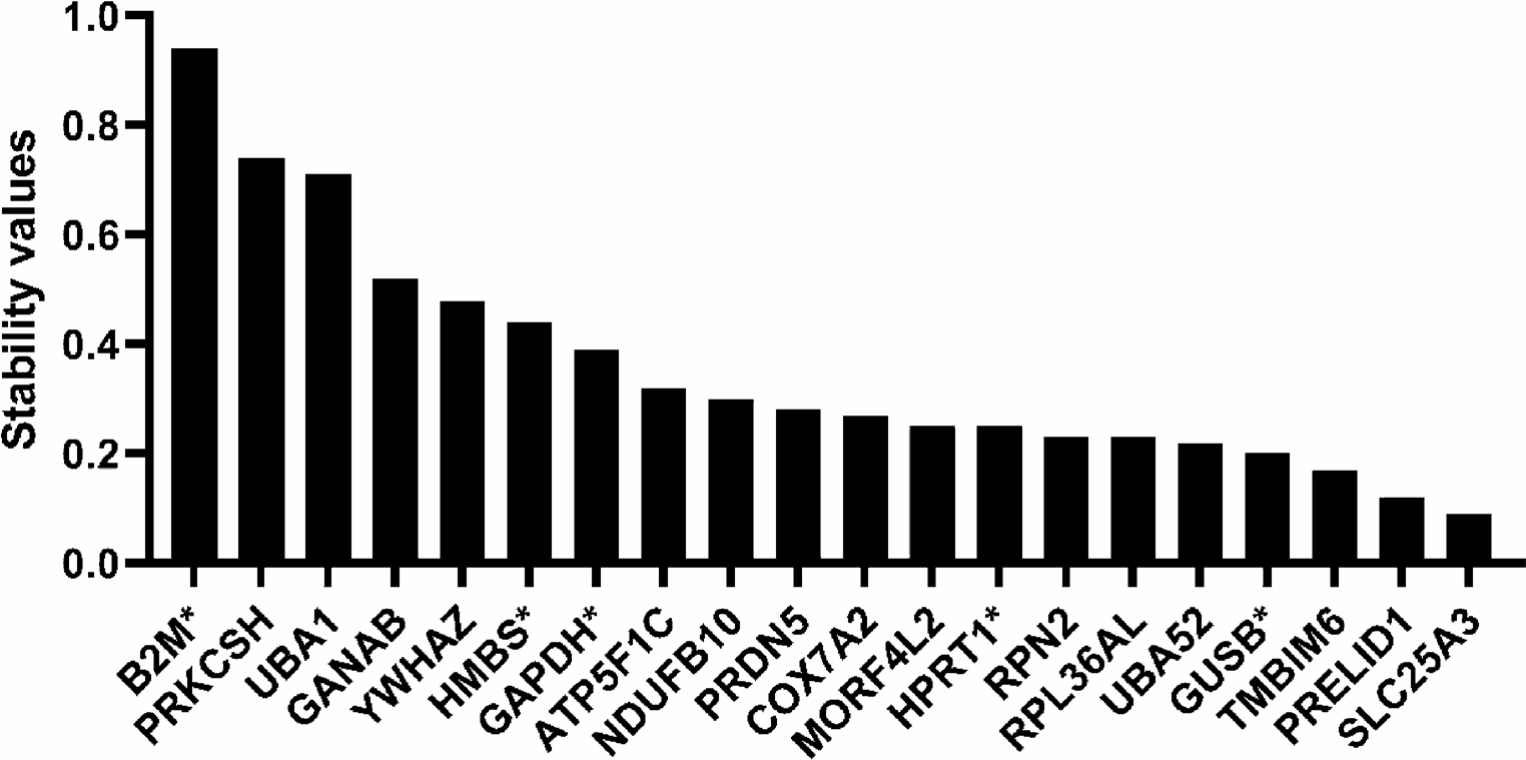
*NormFinder* ranking based on stability value. Housekeeping genes are plotted based on their stability value from the least (left) to the most stable (right). The five added common housekeeping genes are denoted with an asterisk.

The original Article has been corrected.

## Supplementary Information

Supplementary Information.

