# Supplementary material for: Author Correction: Identification of stable housekeeping genes for induced pluripotent stem cells and -derived endothelial cells for drug testing
Source: Sci Rep. 2025 Jun 16;15:20071. doi: 10.1038/s41598-025-04721-x (PMC12170879; doi:10.1038/s41598-025-04721-x)
Supplement: Supplementary file 1 — Supplementary Information. [file 41598_2025_4721_MOESM1_ESM.pdf]

Identification of stable housekeeping genes for induced pluripotent stem cells and -derived endothelial cells for drug testing

Sheena L. M. Ong,[1] Hans J. Baelde[1], David G.P. van IJzendoorn[1], Judith V.M.G. Bovée[1] and Karoly Szuhai[2]*

Supplementary information

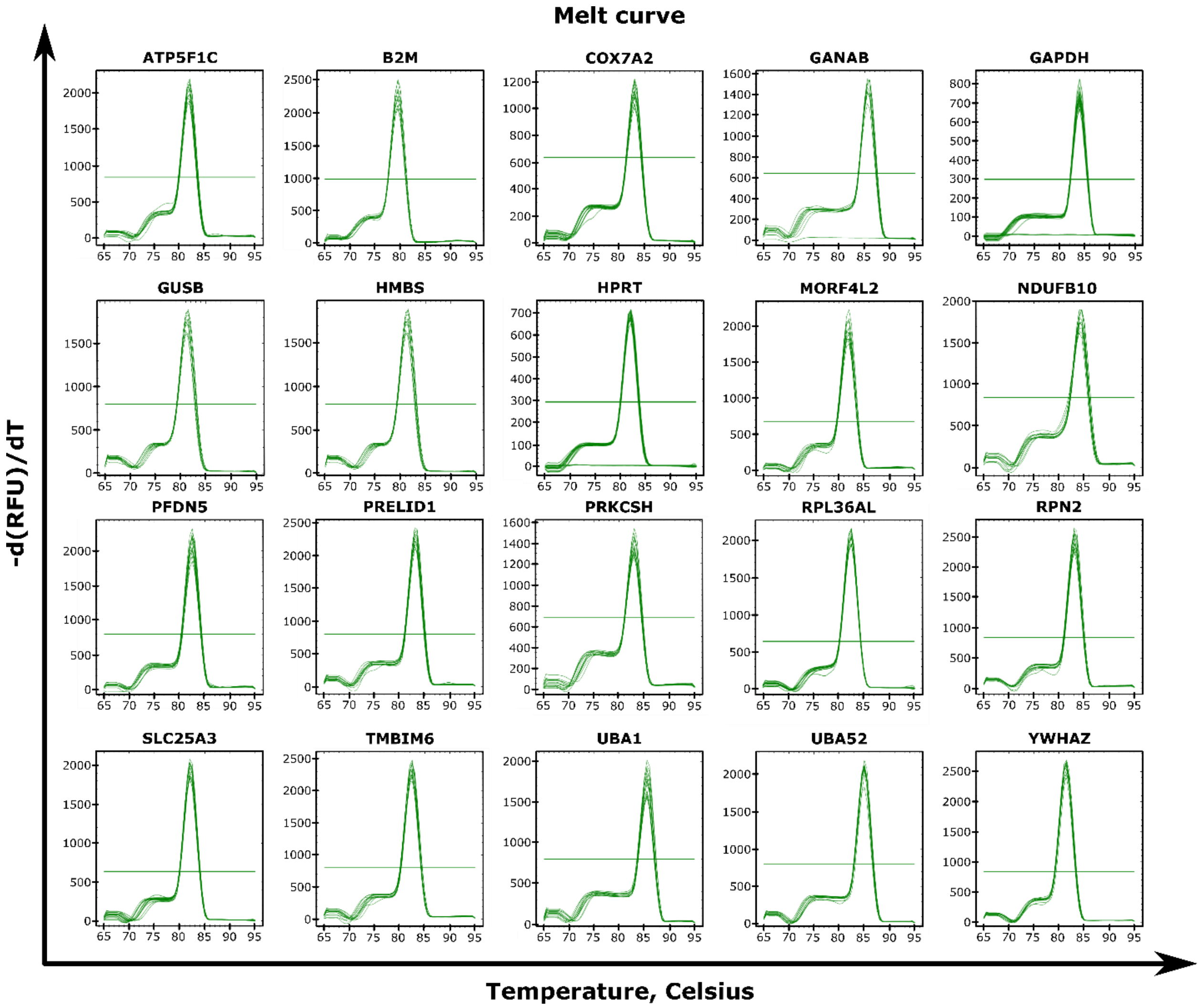

Supp Figure 1. Melt curve of selected housekeeping genes. Melt curves of 20 housekeeping genes were plotted using serial dilutions of qRT-PCR were run on each primer pair. All 20 housekeeping genes shows a high specificity of primers as all of their melt curve peaks at the same point.

Supplementary Table S1. Mean Ct, STD and CV% of 20 HKG

Gene	Mean Ct	STD	CV(%)
COX7A2	21.54	0.30	1.40
HPRT1	24.51	0.36	1.47
YWHAZ	20.52	0.37	1.79
PRDN5	21.34	0.42	1.94
RPL36AL	19.09	0.42	2.21
ATP5F1C	21.80	0.48	2.22
UBA52	18.99	0.49	2.61
NDUFB10	22.18	0.53	2.38
PRELID1	21.37	0.59	2.76
MORF4L2	20.70	0.60	2.90
SLC25A3	20.55	0.63	3.05
GUSB	23.02	0.66	2.86
TMBIM6	21.31	0.71	3.33
GAPDH	19.61	0.88	4.47
RPN2	21.40	0.95	4.44
HMBS	25.52	1.02	3.99
GANAB	24.72	1.09	4.39
B2M	22.32	1.11	4.96
UBA1	25.08	1.66	6.60
PRKCSH	25.49	1.78	6.98

Supp Table 2. Housekeeping genes and primer sequences

Gene	Name	Accession Number	Forward primer sequence	Reverse primer sequence	Amplicon size (bp)	R^2
**ATP5F1C**	ATP synthase F1 subunit gamma	NM_001001973.3	TGAGCAGAGTGCCAGGATGACA	TGTGATGACAGCTTGGCGGGTA	109	1.00
**B2M**	Beta-2-Microglobulin	NM_004048.4	CTATCCAGCGTACTCCAAAG	TCAATGTCGGATGGATGAAA	109	1.00
**COX7A2**	cytochrome c oxidase subunit 7A2	ENSG00000112695	TGGTCAGTAACAGCCAAGATG	TTTTAAAATGCCTGCGGGAAG	94	1.00
**GANAB**	glucosidase II alpha subunit	NM_198334.3	GCTTGGCTTCTAAGAGGCGGAA	CAGCCCTCATAGTCAGAGCCAT	139	0.99
**GAPDH**	Glyceraldehyde-3-Phosphate Dehydrogenase	NM_002046.7	GAGTCAACGGATTTGGTCGT	TTGATTTTGGAGGGATCTCG	238	1.00
**GUSB**	Glucuronidase Beta	NM_000181.4	ACCAGGATCCACCTCTGATG	GAAATCGGCAAAATTCCAAA	128	1.00
**HMBS**	Hydroxymethylbilane Synthase	NM_000190.4	AGGATGGGCAACTGTACCTG	ATGGTAGCCTGCATGGTCTC	82	1.00
**HPRT1**	Hypoxanthine Phosphoribosyltransferase 1	NM_000194.3	CATTATGCTGAGGATTTGGAAAGG	CTTGAGCACACAGAGGGCTACA	129	0.98
**MORF4L2**	mortality factor 4 like 2	NM_012286.3	CTTGAACCAGCTCTCCCAGGAA	TACTGCCACCATCTCCGTTTCC	121	1.00
**NDUFB10**	NADH:ubiquinone oxidoreductase subunit B10	NM_004548.3	GGAAGGACAGAACTACCAGCAG	ACTTCCTGGCAGAACTGTAGGC	116	1.00
**PFDN5**	prefoldin subunit 5	NM_002624.4	TGTGGAAGCCAAGGACTGTCTG	GAGCACGTGTTCCACATCATGC	121	1.00
**PRELID1**	PRELI domain containing 1	NM_013237.4	GGAGGACTCTATTGTGGACCCA	CAGTCCAGCCACTGTTGTCAGA	131	1.00
**PRKCSH**	protein kinase C substrate 80K-H	NM_001289104.2	GCGTCATCTGTGAGAACACCTG	CGTGCCTTCTTCCAGTCCTCAA	130	1.00
**RPL36AL**	ribosomal protein L36a like	NM_001001.5	ATCGGAAGCAGAGTGGCTATGG	CAGCATCCTCTTGGATCTGCAG	125	1.00
**RPN2**	ribophorin II	NM_002951.5	AGCCACCAGAACTTCGCCTTGT	CGGCAACAAACACCACTTCCTG	127	0.99
**SLC25A3**	solute carrier family 25 member 3	NM_002635.4	CTGGCTCCTATGGAAGCTGCTA	GTCTCATCCAGAGAGGAGCAAC	142	1.00
**TMBIM6**	transmembrane BAX inhibitor motif containing 6	NM_003217.3	GCTGATGGCAACACCTCATAGC	GTTGACAGCAATACAAAACTCCAG	121	1.00
**UBA1**	ubiquitin like modifier activating enzyme 1	NM_003334.4	TCCTCACAGAGGACAAGTGCCT	CTTGAGCAGCTCACAGCCAATG	143	0.99
**UBA52**	ubiquitin A-52 residue ribosomal protein fusion product 1	NM_001033930.3	GCCTGCGAGGTGGCATTATTGA	TTCTTGCGGCAGTTGACAGCAC	124	0.98
**YWHAZ**	tyrosine 3-monooxygenase/tryptophan 5-monooxygenase activation protein zeta	NM_145690.3	ACCGTTACTTGGCTGAGGTTGC	CCCAGTCTGATAGGATGTGTTGG	130	1.00

Commonly used housekeeping genes are highlighted in grey.

Supp Table 3. Other primer sequences

Gene	Forward primer sequence	Reverse primer sequence	Amplicon size (bp)
**FOSB**	AGCAGCAGCTAAATGCAGGA	CCAACTGATCTGTCTCCGCC	71
**SERPINE1**	AGAGCGCTGTCAAGAAGACC	TGAAGTTCTCAGAGGTGCCT	115
**SERPINE1-FOSB**	AGAGCGCTGTCAAGAAGACC	GAAGAGATGAGGGTGGGTTG	223
